# Huntington’s disease caregivers: A qualitative exploration of caregivers experience

**DOI:** 10.1177/13591053251328934

**Published:** 2025-04-17

**Authors:** Síle Carney, Niall Pender, Elaine Rogers

**Affiliations:** 1 University of Limerick, Ireland; 2 Beaumont Hospital, Dublin; 3 Health Services Executive, Ireland; 4 Trinity College Dublin, Ireland

**Keywords:** Huntington’s disease, neurodegenerative disease, informal caregivers, family caregivers, caregiver burden, qualitative

## Abstract

Caring for a family member with Huntington’s disease can be complex and challenging. This study set out to investigate the research question: what are the experiences of Huntington’s disease caregivers. In this qualitative study, 11 adult caregivers (6 female and 5 male, aged 49–77 years), of a family member with Huntington’s disease participated in semi-structured interviews, using either video-link or telephone. Data analysis was informed by interpretative phenomenological analysis. The analysis identified three themes related to the caregiver experience: The impact on the caregiver; The impact of the genetic risk; Accessing health care & support services. Complexities of caregiving are compounded by the lack of services available to Huntington’s disease patients, as well as the lack of knowledge and expertise related to the disease. This study highlights the need for enhanced knowledge of the disease in community services, along with access to psychological and multidisciplinary support.

## Introduction

Huntington’s disease is a neurodegenerative disease, that starts insidiously and progresses over 15–20 years. It is debilitating for patients and heartbreaking for family members, who often act as caregivers. An autosomal dominant neurodegenerative disorder, caused by excessive expansion of CAG repeats in the huntingtin gene (Erkkinen et al., 2018), Huntington’s disease is characterized by deterioration in movement control, cognition and behavioral/psychiatric symptoms (Bachoud-Lévi et al., 2019). A clinical diagnosis of Huntington’s disease is only made when there are motor features of the disease present. However, it is becoming clear that individuals may present with many of the nonmotor symptoms—including cognitive dysfunction and psychiatric symptoms—years prior to the onset of motor involvement (Bates et al., 2015; Erkkinen et al., 2018; Roos, 2010; Tabrizi et al., 2022). Impairment in cognitive functioning may include deficits in executive functions, social cognition and decision making (Erkkinen et al., 2018; Mason et al., 2021). Psychiatric symptoms can include depression, suicidal ideation and increased suicide risk, anxiety, apathy, irritability, aggression, disinhibition, antisocial behaviors, and psychosis (Eddy et al., 2016). Although the cognitive and neuropsychological profiles of the disease are often considered secondary to the motor involvement in Huntington’s disease, arguably, these features can have an enormous impact on both the individual and their families’ lives (Beglinger et al., 2010). There is currently no cure or disease modifying treatment for Huntington’s disease. The focus remains on symptomatic management (Bachoud-Lévi et al., 2019; Erkkinen et al., 2018), however, care may vary based on clinical experience rather than scientific evidence (Killoran and Biglan, 2014).

Caring for a family member with Huntington’s disease can be complex and challenging, perhaps even more so than caregiving in other neurodegenerative diseases (Tarolli et al., 2017). The unique complexities of Huntington’s disease mean that some caregivers provide care across two or three generations while being the only gene negative individual in a family. Carers can also be gene positive and providing care while waiting for the disease to develop in themselves. Both of these situations can cause great distress to caregivers (Domaradzki, 2015). Huntington’s disease caregivers reportedly neglect their own needs and experience reduced quality of life as a result of their caregiving role (Aubeeluck et al., 2012).

Stage of life may be influential, with younger caregivers at greater risk for caregiver burden (Zacher and Froidevaux, 2021). Although people have unique life experiences at different times in their lifespan, there are common stages across the lifespan, for example, in young and middle adult (ages 30–50) people are often focused on relationship, career and family development (Panchal et al., 2017; Zacher and Froidevaux, 2021). At these stages a person may be fulfilling multiple roles, at a time when Huntington’s disease onset is most common. Caregivers may be at greater risk of experiencing stressors related to this life stage (Williams et al., 2012).

There is a significant relationship between Huntington’s disease caregiver burden and neuropsychological symptoms, but not motor scores (Hergert and Cimino, 2021). Compared to caregivers of other neurogenerative diseases, Huntington’s disease caregivers experience greater anxiety and depression, and describe their caregiving role as ‘extremely impactful’ on their emotional wellbeing, disrupting their employment/educational roles, and negatively impacting their friendships (Exuzides et al., 2022).

Much of the extant literature addresses challenges associated with caregiver roles; however, there remains limited research exploring the positive aspects of the role (Domaradzki, 2015; Roscoe et al., 2009). The current study aimed to add to, and inform, this literature, through examining the experience of Huntington’s disease caregivers, the impact of providing care, considering both positive and challenging aspects of the role, and identifying unmet needs.

Qualitative research methods are increasingly popular and influential in psychological research (Chapman and Smith, 2002; Willig and Rogers, 2017). Willig reflects that qualitative research:

is concerned with the quality and texture of human experience and with the ways in which people construct and communicate meaning in social contexts…. (Willig, 2012).

Given the research focus there is a strong rationale for a qualitative approach. Qualitative research facilitates a more sensitive and empathetic discussion of the nuances of the human experience. It provides the opportunity to minimize any stigma that may arise. Previous mixed methods research in caregivers of neurodegenerative diseases reported qualitative data provided a more nuanced understanding of the impact of caregiving (Galvin et al., 2016).

The research question was, what are the experiences, both positive and negative, of family caregivers of Huntington’s disease patients?

## Methods

### Sample and setting

Participation criteria included adult caregivers providing care in the home for a family member with Huntington’s disease. The research was advertised through the Beaumont Hospital Huntington’s Disease research group mailing list and Huntington’s disease events throughout Ireland. Caregivers who had previously expressed interest in participating in research with this group were contacted and invited to participate. When caregivers expressed interest in participating, the formal information sheet was emailed to them along with an electronic consent form for research participation. Information sheets outlined the aims of the research, information related to the study and included the contact information for the principal investigator. Twenty people expressed interest in participating, of which eight people could not be contacted, and one person was excluded as the person they were caring for was in a long-term care facility. A total of eleven caregivers consented to participation, were interviewed, and their data included in the analysis. Participants ranged in age from 49 to 77 years (Average = 60.6 years). Table 1 summarizes the participants demographic information.

**Table 1.** Caregiver demographics.

Age*		Range (mean) 49 – 77 (60.6)
Gender		n (%)
	Female	6 (54.5)
	Male	5 (45.5)
Relationship to patient		*n* (%)
	Spouse / Partner	8 (72.7)
	Parent	3 (27.3)

* Age data are based on *n* = 10 caregivers, *n* = 1 did not disclose their age.

### Data collection procedures

In consultation with clinical experts, Huntington’s disease researchers, researchers involved in other neurodegenerative research groups, and based on relevant literature, an interview guide was created. Individual semi-structured interviews were conducted focusing on the experience of Huntington’s disease caregivers examining both positive aspects and challenges related to the role. The use of semi-structured interviews allowed for the interviewer to engage with the participant in an explorative manner to allow for the examination of broad topics as well as focus on specific issues that arose during the interview. Broad topics included exploration of the participants’ overall caregiving role, their experience with healthcare services, identification of positive aspects related to caregiving as well as challenges and support needs. Table 2 illustrates interview topics and questions.

**Table 2.** Interview questions.

Interview topic	Sample question
Background information	Tell me a little bit about your own caring role and duties.
Psychological support	Have you / do you receive psychological support / counselling? Did/do you find it beneficial? Would it be something you would consider in the future?
Health care services	Do you feel you have access to appropriate health care professionals (HCP’s)? Do you feel you can discuss your experiences with HCP’s?
Needs	What are your needs at the moment? Is there anything that would help at the moment?
Positive & negative aspects of caring	Is there anything positive about caregiving? Are there any difficult aspects to caregiving?

Interview duration averaged 63 minutes (range: 30 to 108 minutes). MS teams was utilized for 9 of the interviews. As a result of internet connectivity issues, telephone interviews were utilized by two caregivers. The first author conducted all interviews and had received prior formal training in qualitative methods. The interviewer had no previous familiarity with study participants.

### Analyses

Interviews were recorded and transcribed verbatim. Interpretative phenomenological analysis (IPA) (Smith et al., 2021) was identified as an appropriate approach to analysis. IPA has three core features: it is phenomenological—concerned with exploring peoples’ experiences; it is interpretative—the researcher seeks to understand how the participant makes sense of their life; and IPA is ideographic—it analyses each case individually to fully understand each person’s experience and meaning making of their situation (Hill et al., 2005).

In the first stage of the analysis the researcher “stays close” to the data and the participants’ accounts. During this initial step the first researcher read and re-read all interviews. The next stage involves taking a “step back” to make sense of the data wherein the researcher began initial coding. The research team formulated the preliminary findings. A dynamic and interactive analytic process was adopted by the research team, and a variety of participant experiences were analyzed case by case, then across cases, to bring forth events and meanings.

A dialogue developed between the researchers, the analyzed data, and our psychological knowledge, in particular our professional experience supporting caregivers, working with disability, dementia and rare diseases, and engaging in qualitative research (Smith et al., 2021). To inform their contributions, the research team had sight of the transcripts and initial coding. The development of the themes was an iterative process. The analysis was discussed and themes were explored and developed in regular research team meetings and through revision of the text.

### Reflexivity

Reflexivity was emphasized throughout. Combined, the research team has extensive research and clinical experience in neurodegenerative disorders and caregivers. Considering researchers’ prior experience with informal caregivers, there was potential to make assumptions or create biased interpretations of data. The use of a reflective diary and discussions with the research team throughout the study facilitated an awareness of biases that may have arisen.

### Ethical considerations

This study was approved by the Beaumont Hospital (Medical) Research Ethics committee (ref: 18/45) and University of Limerick Education and Health Sciences Research Ethics Committee (ref: 2023_01_20_EHS (OA)). All participants provided written informed consent prior to enrolment in the study and to their data being published anonymously. This research was conducted ethically in accordance with the World Medical Association Declaration of Helsinki.

## Results

The analysis identified three overarching themes, with sub-themes, related to the caregiver experience: Theme 1: The impact on the caregiver; Theme 2: The impact of the genetic risk; and Theme 3: Accessing health care & support services. See Table 3.

**Table 3.** Themes and sub themes.

Themes	Sub themes
1. The impact on the caregiver	· Positive experiences · Loss, Loneliness, Isolation · Roles and Responsibilities
2. The impact of the genetic risk	
3. Accessing health care & support services	· Accessing services · Lack of understanding · Medical decision making

### Theme 1: The impact on the caregiver

This theme captures the extent of the impact of caregiving for a family member with Huntington’s disease on the caregiver. Data from all participants contributed to this theme.

#### Positive experiences

Whilst most caregivers denied any positive aspects related to their provision of care in the home, caregivers described conflicting nature of their role, “*I feel positive for doing it … I wish I didn’t have to do*.” Whilst another caregiver described the additional time she can spend with her loved one, who remains in the home because of their care needs, as positive, she also acknowledged her disappointment that her loved one is unable to live their own life outside of the home. Some caregivers responded with absolute certainty, describing the role as “*soul destroying*,” “*it’s a chore*” and “*it’s like a black cloud hanging waiting to burst*,” with no indication of any positive, or acquiring any meaning, from the role. Finally, caregivers indicated that assuming the caregiver role was a responsibility within their relationship, therefore, there was a sense of duty to provide care and support in the home. Of note, one caregiver described the humiliation that they feel related to seeking support “*it’s humiliating trying to get benefits, fighting to get benefits, when people are really sick*.”

There were no other positive aspects of caring identified by the sample. Furthermore, when caregivers were asked what would help them, all caregivers reverted back to the care needs of their loved one.

#### Loss, loneliness and isolation

All caregivers reported that the caregiver role negatively impacted their psychological wellbeing. Caregivers discussed the shock of the diagnosis, and the loss of identity they experienced as the disease progressed and their caregiving role became more consuming.

“I melted away in the background because you know you’re trying to support him all the time, there’s a few things that I was involved at the time that I stopped, courses, we were trying to get our head around the diagnosis at the time and the kids were, our youngest was in school, I was doing my own courses … but the headspace wasn’t there to continue, identity wise you do because you’re, it’s kind of sort of shadowing all the time.” (Wife, 58 years)

“The loss of one’s partner was a poignant finding, witnessing one’s partner becoming less competent within the home, losing presence and influence.”

Most caregivers highlighted the behavioral and cognitive changes as challenging aspects of the disease. They are reported as the symptoms that cause the greatest distress in terms of management and disruption in the home, but also indicate a loss that occurs within the relationship.

“…the behavioural aspects of the condition and there was a lot of friction in the house, there was a lot of anger, that was starting to come to the surface and a lot of rows over futile, silly things that happen with Huntington’s disease patients, you know fixations.” (Husband, 49 years)

Caregivers highlighted the sense of isolation and loneliness that occurs as the disease progresses and their relationship with their loved one changes. This was particularly poignant for spousal caregivers. When speaking about the disease, caregivers spoke about Huntington’s disease and the person as being separate entities; as the disease progresses, they lose more of their loved one and their relationship.

“That’s becoming isolating because to see the lines being blurred between husband and carer and you know, that’s devastating.” (Husband, 49 years)

“It’s the very nature of the illness, it just takes a lot of the dignity away from the whole of a relationship, for want of a better phrase, I don’t know quite what way you’d want to put it, you probably lose, you lose a certain persona of your relationship … usually you lose a lot of intimacy, you don’t have any intimacy, the relationship can be quite cold in some ways and that could be hard at times to deal with.” (Husband, 53 years)

Caregivers reported feeling distanced from emotional support systems; they don’t want to ‘bore’ or ‘burden’ friends or family. Caregivers reported feeling that if they described their reality, people wouldn’t enquire again. Instead, they rely on scripts.

“People say ‘how are you?’ And all they want is ‘Yeah, we’re fine, thanks’. They have asked you then … I’m asked every other week how’s Paddy? ‘Great, and very good for what he has’, I say, ‘he’s doing very well’, and that’s that. If I sat down and got into the gritty details of my last two or three weeks and all the different things that happen to me during the week … they would not want to see me next week and say how is Paddy! So now they can happily ask me ‘how is Paddy’ and I can say my small spiel (script).” (Wife, 69 years)

“People want to be interested to know what’s going on, but like you feel if you start to tell them, you’re, kind of maybe, looking at them thinking, ‘you’ve regretted asking me’ … because it is all sorts of unpleasantness you know … So you can just think, well, your course of action is just not to share, just keep on keeping on, put the smiles on, ‘everything’s OK, Jane’s doing great’, you know and you come out with the scripts every day.” (Husband, 49 years)

This sense of isolation was fueled, in part at least, by the lack of understanding of the disease within the wider community. Most caregivers reported that it’s easier not to ask for help, to limit having to constantly educate people on the reality of the heterogeneity in Huntington’s disease presentations.

“There’s been a few times where you know you think you’re reaching breaking point, you know, where it’s got really, really rough and you kind of do feel, you know, the loneliest person in the room because there’s very few people that you can talk to, [for] a very simple reason, it’s a very difficult condition to explain to people, it’s like motor neurons illegitimate brother or illegitimate sister, nobody wants to talk about.” (Husband, 49 years)

#### Roles and responsibilities

Most caregivers reported an increase in roles and responsibilities within the home, previously shared with their loved one. Caregivers reported difficulty with the gradual increase in care needs and demands within the house, both from the perspective of witnessing the decline in functioning experienced by their loved one, and the increased care burden they experienced.

“I wouldn’t say that we co-parent anymore, he doesn’t really parent as such … so if we’re talking about a parent-teacher meeting in school, if we’re talking about paying college fees and doing all those things online, if we’re talking about getting braces at the orthodontist, and any of those things that happen,… he wouldn’t know about them.” (Wife, age undisclosed)

Caregivers in employment identified the stress and worry they experienced as they battled with decisions around their employment, whether they should resign from their positions and provide the level of care they determined appropriate. Whilst considering this, they also highlighted potential financial strain.

“I’ll be faced with just having to give up work, give up everything to care for Mary at home, or, put her in the facility, … Mary’s father refused to put her [mother] in the facility because he said she would have been dead within a year. And you know, you’re battling all this and you’re thinking I’ve two teenagers you know, I’ve got to keep the lights on, the roof over the heads, food on the table, you know, I can’t walk away, you know, how easy it is for me to walk away from the job? I think about it every month you know, how could I make it work? And the only answer is, I can’t, you know, and these are the sort of things that keep you awake at night” (Husband, 49 years)

Of note, caregivers acknowledged that their employment was a source of meaning and purpose in their lives, therefore, there was a risk of losing this also.

“I suppose that’s important you know that I do keep working and that is a sense of identity for me … So it’s a sense of purpose, and I definitely would miss that if I didn’t have it. Plus, I think when you don’t have much interaction, conversation, stimulation at home, like my husband is not the man I married in terms of social interaction, stimulation and things that we would have had in common, so you need to get that somewhere and I get some of that in work.” (Wife, age undisclosed)

### Theme 2: The impact of the genetic risk

Data from nine of the eleven participants addressed the genetic component of Huntington’s disease, acknowledging both the difficulty of having seen other family members progress through the disease, and the fear that their loved ones might present similarly.

“Jack started showing symptoms when I met my husband, that was 30 odd years ago. He was his kid brother, a few years younger than him. … I used to think he was on drugs… he started doing weird things … I started noticing things like that, spending, … then going weird in relationships too, you know … his movements started going mad … he was banging his head off walls, not intentionally, the body moving itself … because it’s so horrific and I saw it last for so long, and how horrific it actually is and like are my children going to be like this?” (Mother, 61 years)

Uncertainty around disclosure with children and the potential impact on their relationships was reported.

“We wanted their paths to be normal in what they wanted to do and not be tainted I suppose or gone off in a different direction because of potentially having the gene themselves, so we wanted them to take their 20’s, map the way that they were going to go themselves and then give them the information and we don’t know if it was right, if it’s the right thing to do, we may have serious fallout over it, but that’s the decision we made at the time.” (Wife, 58 years)

Caregivers highlighted that family members were reluctant to get tested and those who had been tested were reluctant to access results. Caregivers reported hypervigilance for signs and symptoms presenting in their children.

“My other son has the test done but he hasn’t got the result, he doesn’t want it yet, he does want it one minute, he is saying he’s having problems.” (Wife, 51 years)

“I do worry about the kids, I do worry what the future holds for them, I know the last day we were in [hospital] the chap we were with … he did give me a contact for [Hospital] in relation to if the kids wanted to be tested that they could start the process or whatever or at least start talking to somebody in relation to that … So, Barry is 20, James has just gone 18 there in November and there are some days I think they have something going on and then there’s other days I think they’re the brightest lads on the planet. It is a concern in the back of my head and a bit of me is of the opinion what you don’t know doesn’t worry you and just avoid it for as long as you can, I don’t know, I don’t know like it’s, it doesn’t bear thinking about.” (Husband, 53 years)

Caregivers highlighted the stigma, shame and guilt arising because of the genetic risk related to Huntington’s disease.

“It’s the stigma around the condition that shouldn’t be there, but you know, I need to try and protect them [daughters] you know it’s a hereditary condition they both have a one in two chance of inheriting the condition so you know today’s world being today’s world is, you know you have 2 girls .. they’re only 16 and ‘what were you thinking?’, … ‘why did you have kids?’. … that’s part of the reason why people hold themselves back. I think when it comes to Huntington’s disease, particularly anybody with families and kids, because it’s not like MND (motor neuron disease) … it’s not like Parkinson’s, it’s completely random. It’s the hereditary nature of the condition which creates the stigma, which creates the questions, which creates the ‘why this should be eradicated but it isn’t’.” (Husband, 49 years)

Caregivers worried about the impact of the genetic risk on their children, for example, in terms of accessing health insurance.

“What are the knock on effects, how would it affect the kids you know, even down to what’s the situation regarding insurance you know, car insurance and motor insurance, house insurance or life insurance?” (Husband, 49 years)

### Theme 3: Accessing health care and support services

All participants in this study contributed to this main theme. All participants also reported a sense of feeling “lucky” when General Practitioner’s (GP’s) and other health care providers (HCP’s) meet their needs.

#### Lack of knowledge and understanding

Most caregivers identified the challenges associated with accessing care for their loved one in the Irish health care context. Caregivers highlighted the lack of knowledge and understanding of Huntington’s disease amongst HCP’s.

“It just seems that there is so limited (a) knowledge, (b) expertise and (c) actual physical on the ground supports that people could realistically tap into, and anything that we’ve had to tap into, we’ve had to go and search out and seek ourselves, we have to pull all the threads together.” (Husband, 49 years)

“Even doctors, nurses here, especially where we are, there is no, you know, they don’t get it. And I feel as if maybe I might be bothering people.” (Wife, 77 years)

“We’ve great health centres, we’ve everything with fantastic buildings, we haven’t a body inside that knows a thing. Anytime I ever go into my health centre I ask them, ‘do you know what Huntington’s is?’, ‘No, never heard of it’. He has seen the dietician over there … and she doesn’t know absolutely anything at all about Huntington’s, I asked her. She said, no she doesn’t.” (Wife, 69 years)

#### Accessing services

Most caregivers described accessing healthcare from different services depending on their location: including, primary care services, adult disability services, adult mental health, and old age psychiatry. They reported attending irregular neurology appointments (i.e. 6-, 9-, or 12-month appointments). Short blocks of care were discussed as concerning, as they don’t address the evolving presentation of a progressive neurodegenerative disease.

“Yes, we had physio but … they only get physio for certain amount of time … took him to the Huntington’s appointment and he just raised the issue again about more physio because he thought Ronan does benefit from it … so the physio rang me there the other day and said ‘Hi Sinead just checking up, got another letter from your doctor’ and I said ‘I know he’s only allowed so much’ and he goes ‘you know what Sinead, don’t worry about it, I’ll give him another round’. … But unfortunately, like everything else, once you get this round again, I probably have to go back to the doctor again in a few months …. And then you’re still taking the place that somebody else might need, but in the same respect you need it too you know.” (Mother, 55 years)

“The occupational therapist, we were told there is a 2-year waiting list. The dietitian was moved last May, no dietician replaced her in the area I’m in … public health nurse you don’t see her that often, maybe can ring her, but I mightn’t want to, but she doesn’t call and say, ‘how are you getting on, do you need anything?’ So I don’t feel that support is out there for me at the moment, it’s a bit hit and miss.” (Father, 77 years)

Most caregivers discussed the need for practical support such as home help, respite services, appropriate long-term care facilities, and psychosocial support in the form of socialization for their oved one.

“… for me peace of mind would help … would be to know when I go out to work that somebody would have sat with her for an hour at some stage, would have come in to spend time with her.” (Husband, 53 years)

“There’s no home care either, … He would definitely need somebody like social prescribing or befriending or something like that, that would be so useful to somebody at James’s stage or even before. He will need home care at some point and that’s probably coming in the next 12 months or so, but who knows, because we don’t know, I suppose the social prescribing or the befriending, if they could be in place which would lead into a home care package, you know it’s not a case of waiting until you can’t wash, dress or eat before you get home help, there’s so much work in the lead up to that, like we have 5, 6, 7 years of deteriorating functionality and socialisation to be addressed so, but that’s definitely not available or has never been offered.” (Wife, age undisclosed)

“I do need, sometimes maybe, a little more, he did go once for respite care because I was in hospital. I do you need respite care because I’ve a sister who has got stage four cancer and I can’t get to see her.” (Wife, 77 years)

#### Medical decision making

Finally, all caregivers reported difficulty discussing their experience of the patient’s presentation and functioning with HCP’s when their loved one is present at the appointments. Caregivers reported that the patient may not provide the HCP with the most accurate information, and consequently, the needs of the person with Huntington’s disease may not be met. Furthermore, caregivers reported that they are the ones who truly understand the experience and the presentation of the patient in day-to-day life.

“There was very much, and rightly so, the focus on James himself, but very little interrogation of the data that he was presenting, so if he was sleeping well, eating well, bubbly, outgoing personality, I mean, there wasn’t really scope in that consultation for family member input which wouldn’t have been completely different, but certainly there would have been a 30 to 40% difference in terms of how James himself was presenting. Now I suppose I did manage to get some of that information out there and we got medication to reflect that, which has significantly improved his life at home, his sleep and maybe his aggression in the home, but had I not been there or had I not been assertive enough to vocalise what I see in the home, or be able to put words on that well, that may not have been addressed that day. So I suppose the challenge is that it’s very much patient focused which is fine, but we know that Huntington’s affects the family. So there wasn’t, I didn’t feel there was scope, bar I was assertive enough, to get it across and I would be concerned that other family members might not have the skills or resources to push that … .” (Wife, UD)

“Obviously Ronan is capable of discussing his own issues himself right now, so they’re not really looking to me for information per se, but if I feel, that there’s something that should be happening, like say the counselling then I am going to say ‘no’ you know, he’ll say he’s fine, but he ain’t living with him, I’m living with him you know, he needs whatever the case may be, so yeah, you do have to be forceful, I find that with a lot of hospital appointments, you know, you need to stand your ground, if you don’t talk up, they’re just, it’s a tick box exercise.” (Mother, 55 years)

Finally, the impact of disease symptoms, and emotional, relational and pragmatic concerns, including worry about access to insurances and mortgages, were interwoven in participants’ narratives across themes.

## Discussion

Neurodegenerative diseases are becoming more common and a growing cause of mortality internationally (Erkkinen et al., 2018). There is an increasing awareness of the positive societal impact of caregivers providing care in the home (AARP, 2020). Despite an established research base, there remains limited supports in place to help Huntington’s disease caregivers in their caregiving roles. The current research aimed to explore this subgroup of the population, to identify needs, reduce the knowledge gap and inform service development.

This study qualitatively explored the experiences of 11 Huntington’s disease caregivers in Ireland. Three themes were identified through a process of IPA; (1) the impact of the role on the caregiver; (2) the impact of the genetic risk; and (3) access to health care and support services. The findings of the current study indicate the experience of caregivers is characterized by competing pressures, worry about other family members, the need for advocacy, but the overarching experience is an accumulation of losses, loss of identity and sense of self, loss of companionship, intimacy, partnership, and loss of connection with wider social supports (Leidl et al., 2023; Williams et al., 2012). Furthermore, stigma, shame, worry, and secrecy characterize the hereditary nature of Huntington’s disease creating a psychological burden for caregivers. There was very little emphasis on positive experiences of the caregiving role.

Compared with other groups of caregivers, the present sample of Huntington’s disease caregivers, identified few positive experiences of caregiving (Lloyd et al., 2016). The complexity of caregiving in Huntington’s disease—the variation in presentation, and then care needs, of Huntington’s disease patients, along with the genetic risk, creates an environment where it appears that caregivers struggle to identify their positive aspects of their caregiving role (Mackenzie and Greenwood, 2012), in the way that other groups of caregivers can. This has implications for the quality of life of caregivers.

Caregivers discussed the emotional impact of the transition from spouse to carer, as well as the increase in roles and responsibilities. The increased demands mean caregivers lose time and space for their own interests, and reflect the progression of the disease and the losses experienced by the patient. In line with the life stage model this is significant for caregivers in middle adulthood, as they identified the number of once shared responsibilities, for which they are now solely responsible (e.g. parenting, employment, household management) (Zacher and Froidevaux, 2021). Caregivers discussed tensions between staying in employment and financially supporting their family, or resigning and caring for their loved one full-time, albeit potentially in financial instability (Domaradzki, 2015).

This study highlighted caregivers experience of symptoms of Huntington’s disease, specifically social, cognitive and behavioral difficulties as most challenging. This is consistent with the extant literature and may be related to the unpredictability of symptoms (Burke et al., 2018; De Wit et al., 2018; Hergert and Cimino, 2021; Kim et al., 2021; Schumacher-Kuiper et al., 2021). Caregivers in this study reported that these symptoms related to a change in the person they once knew and changed the nature of their social interactions (Mason et al., 2021). Caregivers experience prolonged, and sometimes disenfranchised, grief, and ambiguous loss: the breakdown or loss of relationships whilst also managing loved ones’ care is a difficult emotional space to inhabit (Ressler and Gershfeld-Litvin, 2023). Caregivers are managing these transitions and demands whilst also reporting limited emotional support from family or friends: they don’t want to “burden” or “bore” peers. The reluctance to talk about the disease may arise from the adoption of denial tactics to cope, which may contribute to the isolation experienced by individuals with Huntington’s disease and their families (Lowit and Van Teijlingen, 2005).

This study highlighted a unique aspect of Huntington’s disease: the significant genetic risk that exists. Caregivers described experiencing distress when they observe symptoms in family members with Huntington’s disease and worry whether other family members—or indeed they themselves, will present similarly. There is significant distress related to the genetic risk faced by offspring and future generations, with reported feelings of worry, shame and guilt (Williams et al., 2012, Domaradzki, 2015, Lowit and Van Teijlingen, 2005). To protect their children, caregivers choose to withhold information from both their children and the outside world (Van Lonkhuizen et al., 2023). However, it was suggested in the current study that withholding information may be contributing to the prevailing stigma associated with Huntington’s disease. One caregiver suggested Huntington’s disease was “MND’s illegitimate brother,” suggesting the lack of public information and support may be directly related to the stigma around the disease.

The present study highlighted the lack of co-ordinated multi-disciplinary services available to Huntington’s disease patients, as well as the lack of knowledge and expertise related to the disease. Huntington’s disease caregivers’ quality of life is impacted by difficulty accessing healthcare services (Skirton et al., 2010; Soltysiak et al., 2008). Caregivers in the current study highlight the challenges engaging with HCP’s with limited knowledge and accessing supports that do not meet patient needs. Whilst a short-term model of care may prove appropriate for some symptoms of Huntington’s disease, it may not work for every symptom or presentation.

### Clinical implications

This study highlighted the need for socialization for this patient group. Huntington’s disease patients often resign from their employment at a younger than anticipated age, losing access to social networks. Additionally, people with Huntington’s disease may struggle to adjust to the need for personal assistance. Many patients have social cognitive deficits that cause interpersonal and social difficulties making it difficult for caregivers to provide support and often alienating the family from social and healthcare supports (Mason et al., 2021). Introducing supports within the home such as personal assistants earlier in the disease trajectory may be beneficial in terms of both socialization and adjustment to personal care supports when required.

To support both Huntington’s disease patients and their family caregivers, there is a need for specialist care from a multi-disciplinary approach (Bachoud-Lévi et al., 2019; Novak and Tabrizi, 2010; Phillips et al., 2008; Simpson and Rae, 2012). An important finding in this study was the perceived lack of knowledge of Huntington’s disease amongst HCP’s. There is an onus on HCP’s to upskill when presented with unfamiliar disease presentations. There is a role for experts in the field to support upskilling within community services. There is a role also for HCP’s to actively reduce the stigma associated with Huntington’s disease.

Because of difficulties with cognition, caregivers described that the person with Huntington’s disease may not present an accurate medical history to their medical team (Mason et al., 2021). Caregivers described that their collateral information was essential in the successful treatment of the Huntingon’s disease patient. This gives rise to ethical dilemmas including: who is the patient and who needs support? the discrepancy between proxy report, patient report and direct observation; the impact of stress on perception of behaviors as challenging; decision-making capacity and consent to treatment; the role of self- and caregiver advocacy (Kroenke et al., 2022).

Access to psychological support may educate caregivers to support their loved ones with neuropsychological symptoms and apply psychological techniques to support behavioral symptoms. Psychological and social care support may help Huntington’s disease families to manage the distress caused by the genetic risk, supporting the family through counselling, information and open honest dialogue to reduce miscommunication and disinformation. Family support in this manner may tackle the secrecy and stigma caregivers describe arising from the hereditary component of the disease. Caregivers should be supported to maintain their social networks as health-related quality of life has been significantly associated with higher perceived social support (Van Lonkhuizen et al., 2023). This is particularly important in terms of the longevity of the Huntington’s disease caregiving role.

While not exclusive to this population, Huntington’s disease and its management in the community, shines a light on the social contract of caregiving, its relational nature, the need for systemic approaches to treatment and support, and the toll caregiving takes on caregivers (Brennan et al., 2023, Ring et al., 2024)

### Research implications

This research contributes to the evidence base on supporting Huntington’s disease patients, caregivers and their families. Qualitative data is an appropriate method for exploring the nuances of the human experience. Similar research discusses the benefits of utilizing qualitative methods and gaining a more nuanced understanding of the impact of caregiving when compared with quantitative methods (Galvin et al., 2016). Next steps could include exploring cultural perspectives in caregiving, expanding the participants in included samples, and exploring supports for caregivers (Han et al., 2021; Smedley & Coulson, 2021).

### Limitations of this study

The sample in this study reflects some limitations. The sample did not include any adult child caregivers to a parent with Huntington’s disease, or to a sibling with Huntington’s disease. This population did not engage with the recruitment process, but their future inclusion could perhaps bring developmental perspectives to our understanding of caregiving in Huntington’s disease.

The age range of the sample (49–77 years) could be a potentially limiting factor. We earlier discussed the life stage model and the significance of caregiving for adults in middle adulthood (30–50 years). While our sample overlaps with the upper end of that age band, younger caregivers were not represented (D’Amen et al., 2021, Fleitas Alfonzo et al., 2022).

The sample comprised White Irish participants and perhaps is not reflective of the growing diversity in Ireland. The cultural dynamics of caregiving and help seeking were not explored. Neither was stigma explored outside of the lens of the culturally dominant frame. Whilst this study aimed to capture a varied experience of Huntington’s disease, there may be cohorts of caregivers, both described here and others, who did not participate, and their perspectives may not be represented.

Huntington’s disease presents with challenges for patients and caregivers, and demands of HCP’s, providers, researchers, and wider community, a compassionate and coordinated response.
